# Analyses of mRNA Profiling through RNA Sequencing on a SAMP8 Mouse Model in Response to Ginsenoside Rg1 and Rb1 Treatment

**DOI:** 10.3389/fphar.2017.00088

**Published:** 2017-02-27

**Authors:** Shuai Zhang, Dina Zhu, Hong Li, Haijing Zhang, Chengqiang Feng, Wensheng Zhang

**Affiliations:** ^1^Beijing Area Major Laboratory of Protection and Utilization of Traditional Chinese Medicine, Beijing Normal UniversityBeijing, China; ^2^Engineering Research Center of Natural Medicine, Ministry of Education, Beijing Normal UniversityBeijing, China; ^3^Department of Chinese Medicine, College of Resources Science Technology, Beijing Normal UniversityBeijing, China; ^4^National and Local United Engineering Research Center for Sanqi Resources Protection and Utilization TechnologyKunming, China

**Keywords:** Ginsenoside Rg1, Ginsenoside Rb1, Alzheimer's disease, SAMP8, gene-level, RNA sequencing

## Abstract

Ginsenoside Rg1 and Rb1 are the major ingredients in two medicines called QiShengLi (Z20027165) and QiShengJing (Z20027164) approved by China. These ingredients are believed to mitigate forgetfulness. Numerous studies have confirmed that GRg1 and GRb1 offer protection against Alzheimer's disease (AD), and our morris water maze (MWM) experiment also indicated that GRg1 and GRb1 may attenuate memory deficits in the 7-month-old SAMP8 mice; however, comprehensive understanding of their roles in AD remains limited. This study systematically explored the mechanism at the genome level of the anti-AD effects of GRg1 and GRb1 in a senescence-accelerated mouse prone 8 (SAMP8) model through deep RNA sequencing. A total of 74,885 mRNA transcripts were obtained. Expression analysis showed that 1,780 mRNA transcripts were differentially expressed in SAMP8 mice compared with the SAMP8+GRg1 mice. Moreover, 1,066 significantly dysregulated mRNA transcripts were identified between SAMP8 and SAMP8+GRb1 mice. Analyses according to gene ontology and the Kyoto Encyclopedia of Genes and Genomes revealed that oral administration of GRg1 and GRb1 improved the learning performance of the SAMP8 mouse model from various aspects, such as nervous system development and mitogen-activated protein kinase signaling pathway. The most probable AD-related transcriptional responses after medication were predicted and discussed in detail. This study is the first to provide a systematic dissection of mRNA profiling in SAMP8 mouse brain in response to GRg1 and GRb1 treatment. We explained their efficacy thoroughly from the source (gene-level explanation). The findings serve as a theoretical basis for the exploration of GRg1 and GRb1 as functional drugs with anti-AD activity.

## Introduction

Alzheimer's disease (AD) is a progressive amnesic disorder characterized by age-dependent memory loss and impairment of cognitive functions (Yaari and Corey, 2007; Roy et al., 2016). According to reports, two types of AD exist. Sporadic AD accounts for 90–95% of all AD cases, and the familial form of AD accounts for 5–10% (Bhat, 2010; Babusikova et al., 2011). Current statistics indicate that a person in America develops AD every 67 s. By 2050, a new case of AD is expected to develop every 33 s, and nearly 1 million new cases per year is expected to arise (Alzheimer's Association, 2015). AD is becoming a major public health problem, and discovering effective drugs for the treatment, relief, or prevention of AD has become increasingly urgent.

Chinese medicinal herbs exert certain effects on the treatment of neurodegenerative diseases (Howes et al., 2003). *Panax notoginseng*, the root of *Panax notoginseng* (Burk.) F. H. Chen, is a well-known traditional Chinese herbal medicine with a long history of usage in East Asian countries (Yan et al., 2014). *Panax notoginseng* contains more than 30 different saponins called Panax notoginsenosidum (PNS). Ginsenosides Rg1 (GRg1) and Rb1 (GRb1) are the major pharmacologically active ingredients of *Panax notoginseng* (Du et al., 2003). As eminent members of PNS, GRg1, and GRb1 are believed to mediate the complex pathological mechanisms of AD. Zhao et al. reported that GRg1 suppresses the signaling transduction pathway of TLR3 and TLR4 and decreases the inflammation factors induced by Aβ 25–35 in NG108-15 cells (Zhao et al., 2014). Scientific evidence shows that GRb1 reverses the changes in several direct or indirect neuroinflammation markers of AD produced by ventricle injection of Aβ 1–42 (Wang et al., 2011). Li et al. concluded that GRg1 treatment affects all three metabolic pathways, and GRb1 treatment affects lecithin and amino acid but not sphingolipid metabolism in AD mice (Li et al., 2015). These studies provided several clues but not detailed insights and comprehensive assessments.

In the present study, the mRNA profiles of the brain of GRg1- and GRb1-treated senescence-accelerated mouse prone 8 (SAMP8) models at 7 months of age were explored through RNA sequencing. The SAMP8 strain is a naturally derived model that demonstrates cognitive, behavioral, and neuropathological alterations similar to those observed in aged humans; it is a plausible model for exploring the complexity of AD (Butterfield and Poon, 2005; Cheng et al., 2014; Kang et al., 2014). This research systematically provided a molecular basis for the therapeutic benefits of GRg1 and GRb1 in AD treatment. Thus, GRg1 and GRb1 may be potential therapeutic reagents for halting or preventing AD progression.

## Materials and methods

### Preparation of GRg1 and GRb1

**Figure 1:**
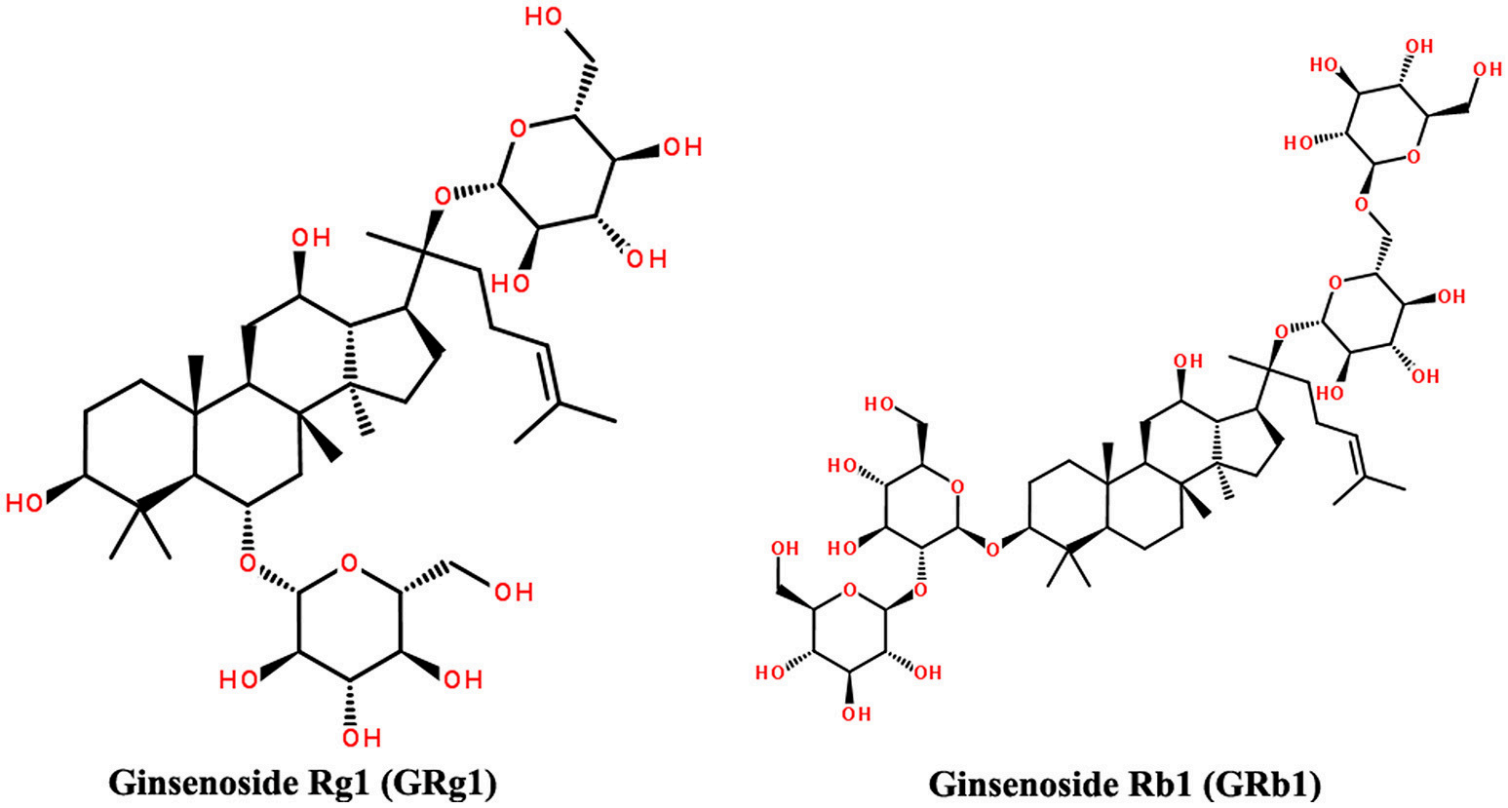
**Structures of GRg1 and GRb1**.

GRg1 (C_42_H_72_O_14_, molecular weight = 801.01, Figure 1) and GRb1 (C_54_H_92_O_23_, molecular weight = 1,109.29, Figure 1) with purity >98% were purchased from the Chinese National Institute for the Control of Pharmaceutical and Biological Products (Beijing, China). GRg1 and GRb1 were dissolved in sterile distilled water before use.

### Animals and grouping

Three-month-old male SAMP8 mice (*n* = 45) were purchased from Beijing WTLH Biotechnology Co., Ltd. Each mouse was housed in a cage in standard specific pathogen-free conditions (24 ± 2°C, 45–55% humidity, and 12 h light/dark cycle). The mice were allowed free access to water and food. The SAMP8 mice were randomly divided into three groups (*n* = 15 mice per group), which received GRg1 (15 mg/kg/d), GRb1 (15 mg/kg/d), and the vehicle (sterile distilled water) via oral gavage. After 4 months of GRg1 and GRb1 administration, three animals were randomly selected from each group and anesthetized to collect the cerebral cortex. The tissues were immediately frozen in liquid nitrogen for RNA sequencing. Eight animals in each group were also randomly selected for the morris water maze (MWM) test.

All of the animal procedures were approved by the Institutional Animal Use and Care Committee of Beijing Normal University and adhered to the “Guide for the care and use of laboratory animals” (Clark et al., 1997).

### MWM test

The spatial learning and memory of the mice were evaluated through the MWM test (D'Hooge and De Deyn, 2001; Vorhees and Williams, 2006). Briefly, in the hidden platform test (days 1–5), a platform was placed at the center of a suppositive quadrant. The mice were subjected to two trials per day for five consecutive days. During each trial, the mice were placed in the maze at four different assigned points and allowed to swim for 90 s. The escape latency was recorded by a software upon mounting the platform. When a mouse failed to reach the platform within 90 s, it was guided to the platform, and the escape latency was recorded as 90 s. In both situations, the mice were allowed to rest on the platform for 15 s and subsequently placed in the home cage. The platform was removed in the spatial probe test (day 6). The mice were released from the opposite quadrant and allowed to swim freely for 60 s. All experiments were conducted at approximately the same time daily.

### RNA extraction and qualification

TRIzol reagent (Invitrogen, Carlsbad, CA, USA) was used to isolate the total RNA of each sample. The purity of RNA was checked with a NanoPhotometer® spectrophotometer (IMPLEN, Westlake Village, CA, USA). The concentration of RNA was tested with Qubit® RNA Assay Kit in Qubit 2.0 Flurometer (Life Technologies, CA, USA). In addition, RNA integrity was assessed with RNA Nano 6000 Assay Kit in the Bioanalyzer 2100 system (Agilent Technologies, Santa Clara, CA, USA). The samples with RNA integrity number scores higher than eight were used in this study.

### Library preparation and sequencing

Nine cDNA libraries were constructed, i.e., three for the SAMP8+GRg1 mice, three for the SAMP8+GRb1 mice, and three for the SAMP8+vehicle mice. We utilized 3 μg of RNA per sample as the input material for RNA sample preparation. First, rRNA-depleted RNA was fragmented with NEBNext first strand synthesis reaction buffer (5X). Second, a random hexamer primer and M-MuLV reverse transcriptase (RNaseH−) were used to synthesize the first-strand cDNA. Second-strand cDNA was synthesized subsequently with DNA polymerase I and RNase H. Third, the purified second-strand cDNA was ligated with NEBNext adaptor after adenylation of the 3' ends of the DNA fragment, and 150–200 bp cDNA fragments were isolated. Then, the cDNA libraries were enriched through PCR amplification with Phusion high-fidelity DNA polymerase, universal PCR primers, and index (X) primer. The Agilent bioanalyzer 2100 system was used to assess library quality. The libraries were sequenced at the Novogene Bioinformatics Institute (Beijing, China) on an Illumina HiSeq 4000 platform, and 150 bp paired-end reads were generated after clustering of the index-coded samples. Clustering was performed in a cBot cluster generation system using the TruSeq PE Cluster Kit v3-cBot-HS (Illumina).

### Transcriptome assembly

Clean data were obtained after filtering out reads with adaptors and poly-N > 10% and low-quality reads from raw data through in-house perl scripts. The Q20, Q30, and GC contents of the clean reads were calculated. All downstream analyses were based on the good-quality clean reads. Paired-end clean reads were mapped to the mouse genome sequence (ftp://ftp.ensembl.org/pub/release-81/gtf/mus_musculus/) with TopHat v2.0.9 (Trapnell et al., 2009; Kim et al., 2013). Cufflinks v2.1.1 was used to assemble the mapped reads of each sample (Trapnell et al., 2010).

### Expression analysis

The protein-coding gene expression levels in each sample were estimated according to fragments per kilo-base of exon per million fragments mapped (FPKMs) and assessed with Cufflinks v2.1.1 (Trapnell et al., 2010). Transcripts with a *P* < 0.05 were considered differentially expressed.

### Quantitative polymerase chain reaction (qPCR)

The results of RNA sequencing were validated through qPCR. qPCR was performed with the LightCycler 480 real-time PCR system and SYBR Green PCR Master Mix (TaKaRa Biotechnology, Dalian, China). The specific quantitative primers for 8 transcripts (four from SAMP8 vs. SAMP8+GRg1 and four from SAMP8 vs. SAMP8+GRb1) are listed in Supplementary Table 1. The 20 μL reaction volume contained 0.5 μL of each primer, 8 μL of H_2_O, 1 μL of cDNA, and 10 μL of 2 × RealMasterMix (TaKaRa Biotechnology). The conditions were 95°C for 2 min followed by 40 cycles (95°C for 20 s, 60°C for 30 s, and 68°C for 30 s). Each experiment was performed in triplicate.

### Gene ontology (GO) and Kyoto encyclopedia of genes and genomes (KEGG) analyses

To understand the functional roles of the differentially expressed protein-coding genes, we used the GOseq R package to conduct an enrichment analysis (Young et al., 2010). GO terms with a *P* < 0.05 were considered significantly enriched. In addition, KEGG enrichment analysis was performed with KOBAS software on the differentially expressed protein-coding genes (Mao et al., 2005) through a hypergeometric test. A hypergeometric *P* < 0.05 was considered significant.

### Statistical analysis

Statistical analyses were performed with SPSS 20.0 software. All data were expressed as the means ± standard error of the mean (SEM). *P* < 0.05 were considered statistically significant. The escape latency in the MWM test was compared through two-way analysis of variance (ANOVA). One-way ANOVA was used to analyze the rest index in the MWM test. Student's *t*-test was used to compare the qPCR results.

## Results

### GRg1 and GRb1 ameliorated memory impairment in 7-month-old SAMP8 mice

**Figure 2:**
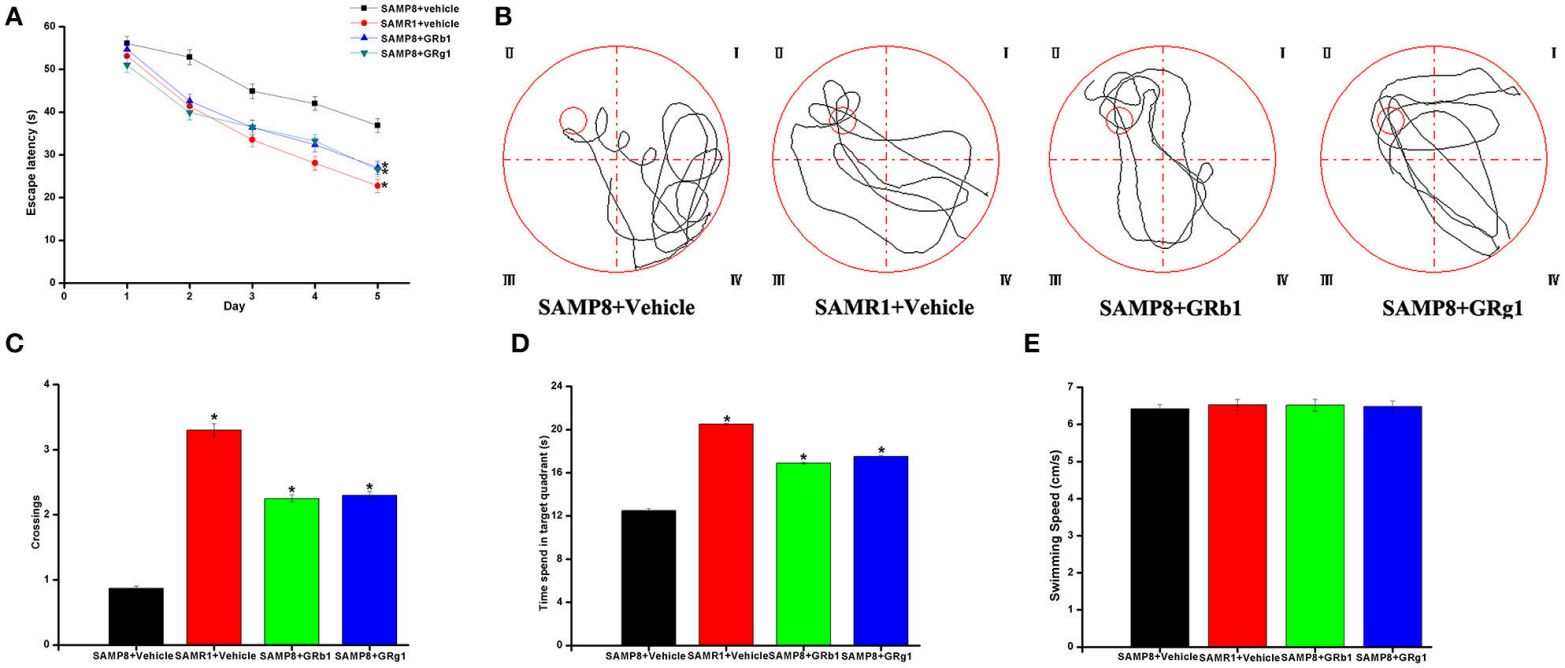
**GRg1 and GRb1 administration ameliorated memory deficit in SAMP8 mice**. Morris water maze tests were conducted on mice with or without GRg1 and GRb1 (15 mg/kg/d, 4 months) treatment. Eight animals in each group. **(A)** Mean escape latency in the hidden platform test. **(B)** Swimming paths in the spatial probe test. **(C)** Number of crossings in the spatial probe test. **(D)** Time spent in the target quadrant during the spatial probe test. **(E)** Average swimming speeds of mice in the visible-platform test. *p < 0.05.

We performed the MWM test to evaluate the effect of GRg1 and GRb1 on the cognitive function of the SAMP8 mouse model. In the result, we introduced the MWM performance of the senescence-accelerated mouse resistant 1 (SAMR1) mouse model based on our previous study (Zhang et al., 2016). The SAMR1 mouse model is widely used as a control strain (Ma et al., 2011). As shown in Figure 2A, the mean escape latency of SAMP8 + GRg1, SAMP8 + GRb1, and SAMR1 mice significantly decreased compared with that of SAMP8 + vehicle mice (*P* < 0.05). In the spatial probe test, the SAMP8 mice swam randomly in the tank without knowing the target location, whereas the GRg1- and GRb1-treated SAMP8 mice and SAMR1 mice searched preferentially for the target quadrant and showed improved memory (Figure 2B). In addition, the number of crossings and the time spent in the target quadrant increased significantly in the GRg1- and GRb1-treated SAMP8 mice and SAMR1 mice compared with the SAMP8 mice (Figures 2C,D). No significant difference was observed in the swimming speed of all the groups (*P* > 0.05, Figure 2E), suggesting that the memory impairments of SAMP8 mice were not caused by motor and visual dysfunctions. Evidently, administration of GRg1 and GRb1 for 4 months significantly attenuated the memory deficit of 7-month-old SAMP8 mice.

### Overview of RNA sequencing

A total of 800,400,106 raw reads (251,783,324 for SAMP8, 277,307,450 for SAMP8+GRg1, and 271,309,332 for SAMP8+GRb1) were generated. After discarding the reads with adapters, poly-N > 10%, and any other possible contaminants, 784,513,910 clean reads (244,770,474 for SAMP8, 272,566,802 for SAMP8+GRg1, and 267,176,634 for SAMP8+ GRb1) were obtained. The clean reads were mapped to the mouse reference genome (ftp://ftp.Ensemble.org/pub/release-83/gtf/mus_musculus/), and the mapping rates were approximately 70.72, 71.41, and 70.06% for the SAMP8, SAMP8+GRg1, and SAMP8+GRb1 mice, respectively. The cufflinks results indicated that 102,250 transcripts were assembled. A total of 74,885 protein-coding transcripts were identified. These mRNAs were used for subsequent analysis.

### Differential expression analysis: SAMP8 vs. SAMP8+GRg1 and SAMP8 vs. SAMP8+GRb1

**Figure 3:**
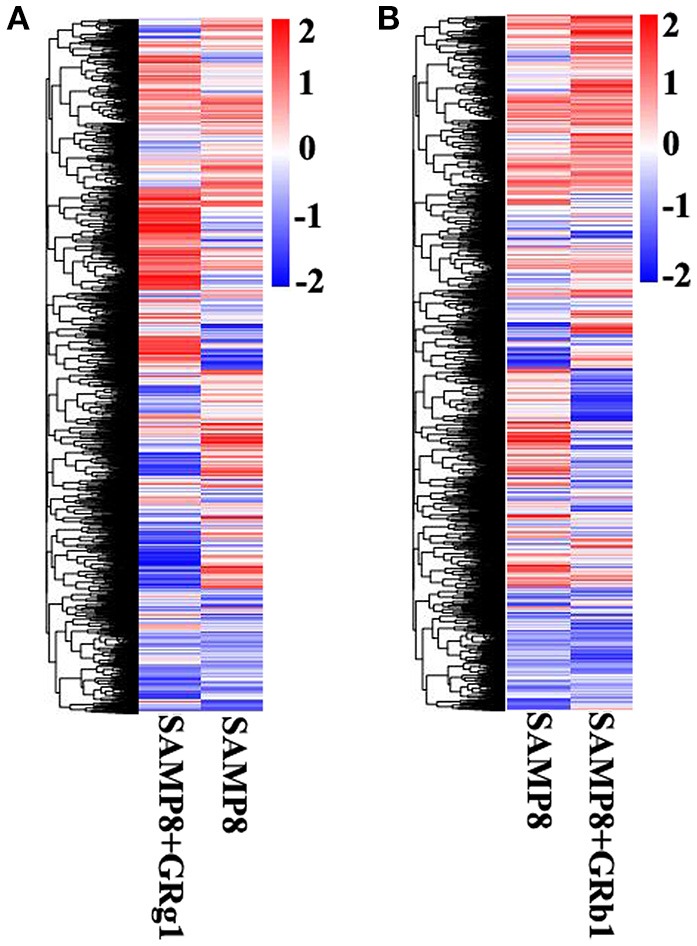
**Cluster analysis by heat map. (A)** Cluster analysis of differentially expressed mRNAs in SAMP8 and SAMP8+GRg1 mice. **(B)** Cluster analysis of differentially expressed mRNAs in SAMP8 and SAMP8+GRb1 mice. The red markings indicate an increased expression, and the blue markings indicate a decreased expression.

The mRNA expression levels and transcripts were estimated with FPKMs. A total of 1,780 mRNA transcripts, including 768 upregulated and 1,012 downregulated transcripts (*p* < 0.05), were differentially expressed in SAMP8+GRg1 mice relative to SAMP8 mice (Supplementary Table 2). Meanwhile, 1,066 significantly dysregulated mRNA transcripts were identified between SAMP8 and SAMP8+GRb1 mice (Supplementary Table 3); 381 were upregulated in the SAMP8 mice, whereas 685 were downregulated in SAMP8+GRb1 mice (*p* < 0.05). Cluster analysis of differentially expressed mRNAs was conducted with heat maps (Figures 3A,B).

### Quantitative assessment

**Figure 4:**
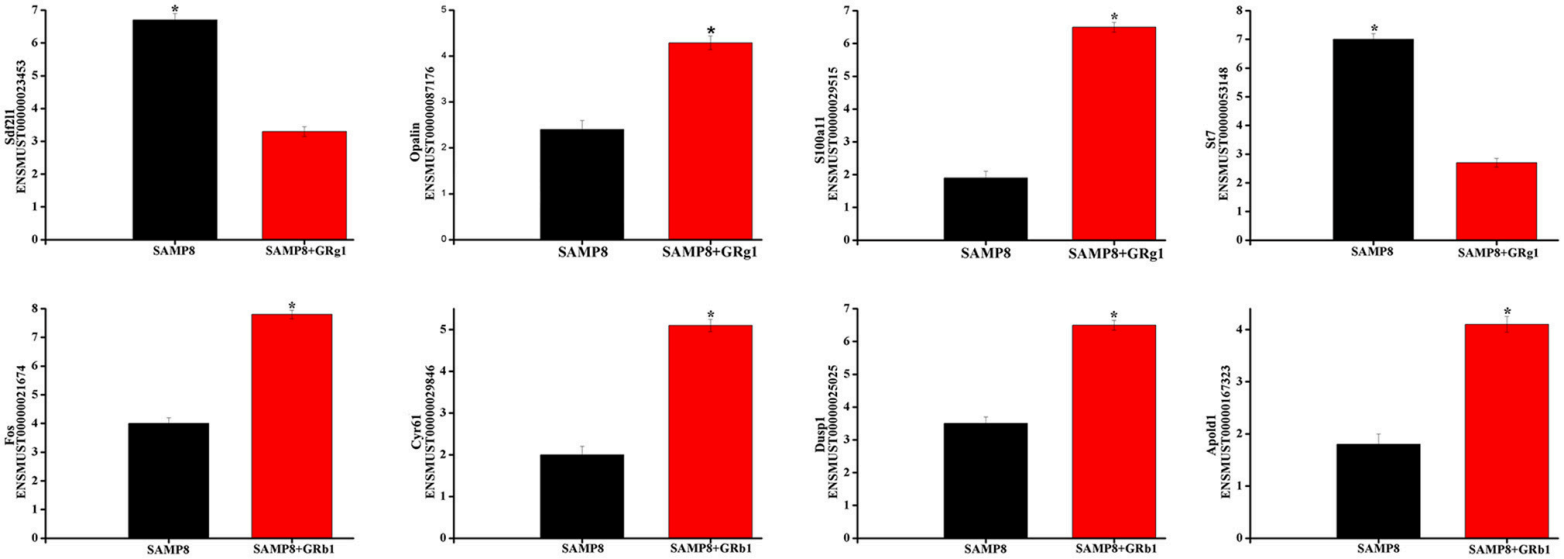
**Validation of transcript expression by qPCR**. Data are presented as the mean ± SE (*n* = 3). β-actin gene was used as a housekeeping internal control. Transcript expression was quantified relative to the expression level of β-actin using the comparative cycle threshold (ΔCT) method. ^*^p < 0.05.

Eight differentially expressed mRNA transcripts (four from SAMP8 vs. SAMP8+GRg1 and four from SAMP8 vs. SAMP8+GRb1) were randomly selected to validate the accuracy of RNA sequencing through qPCR. All the selected mRNA transcripts were detected and exhibited significant differential expressions (Figure 4). These results are consistent with RNA sequencing data.

### Functional annotation

First, GO and KEGG analyses were performed on 1,780 significantly dysregulated mRNAs in SAMP8 vs. SAMP8+GRg1. We derived 1,575 highly enriched GO terms (Supplementary Table 4, *P* < 0.05) and 21 significantly enriched pathways (Supplementary Table 5, *P* < 0.05). We observed several AD-associated pathways and terms, such as protein processing in the endoplasmic reticulum, FoxO signaling, PI3K-Akt signaling, and mitogen-activated protein kinase (MAPK) signaling pathways, and several GO terms (e.g., GO: 0031175, GO: 0007399, GO: 0045686, GO: 0010977, and GO: 0043409). Overall, GRg1 affected AD from different angles.

GO and pathway enrichment analyses were also executed to explore the functions of 1,066 significantly dysregulated mRNAs in SAMP8 vs. SAMP8+GRb1. According to the GO survey, 1,368 GO terms were significantly enriched (Supplementary Table 6, *P* < 0.05). Several terms, including GO: 0000188, GO: 0007420, and GO: 0007416, were closely related to AD. Eighteen significantly enriched KEGG pathways (Supplementary Table 7, *P* < 0.05) were detected. Several of these pathways, including the MAPK signaling pathway, Notch signaling pathway, and cholinergic synapse, were associated with AD. Overall, the pathological process of AD may be regulated by GRb1 from different aspects.

### Comprehensive analysis

In our previous study (Zhang et al., 2016), we identified 2,505 significantly dysregulated mRNA transcripts between SAMP8 and SAMR1 mice. Thus, to further understand the potential protective effects of GRg1 and GRb1 on AD, we set two limiting factors. One was that the gene should be significantly and differentially expressed between SAMP8 vs. SAMP8+GRg1 or SAMP8 vs. SAMP8+GRb1 mice. At the same time, the change in the expression after administrating GRg1 or GRb1 must lean toward the normal group (SAMR1). The other requirement was that the selected gene should be associated with AD. According to these requirements, the fulfilled genes were detected and are listed in Tables 1, 2. For example, *Mapk11* is an enzyme encoded by the *Mapk11* gene (Stein et al., 1997). This kinase is closely related to p38 MAP kinase and is involved in the development of AD. We found that the expression of *Mapk11* in the SAMP8+GRb1 and SAMR1 mice was significantly lower than that in the SAMP8 mice. In addition, an evident reduction was observed in the SAMP8+GRg1 mice, but this reduction was not significant. In short, we predict that GRg1 and GRb1 may regulate the expression of the abovementioned genes and are possibly involved in the adjustment of AD.

**Table 1:** **AD-related genes identified after treatment with GRg1**.

**Gene**	**Transcript ID**	**SAMP8 FPKM**	**SAMP8+Rg1 FPKM**	**SAMR1 FPKM**
Bche	ENSMUST00000029367	0.98	1.33	1.30
Fgfr1	ENSMUST00000084027	1.37	2.21	2.23
Dusp1	ENSMUST00000025025	9.07	10.89	16.53
Cdkn2b	ENSMUST00000097981	0.29	0.52	0.62
Flna	ENSMUST00000114299	3.23	4.64	4.87
Glp1r	ENSMUST00000114574	0.27	0.48	0.77
Slc39a7	ENSMUST00000169397	5.97	1.98	2.59
Calr	ENSMUST00000003912	196.95	159.62	163.60
Mapk11	ENSMUST00000088823	14.36	12.77	11.81
Trpc6	ENSMUST00000050433	22.02	18.76	4.37
Smad3	ENSMUST00000034973	20.09	16.76	16.99
Pdia6	ENSMUST00000057288	85.99	61.29	68.01
Pdia4	ENSMUST00000077290	36.14	24.39	29.83
Xbp1	ENSMUST00000063084	55.75	39.55	42.90
Arntl	ENSMUST00000047321	17.87	13.26	13.49
Gm10053	ENSMUST00000073080	1.79	1.06	0.42
Pfkfb3	ENSMUST00000028114	5.22	3.46	4.07
Htr5b	ENSMUST00000055884	0.85	0.47	0.48

*FPKM, fragments per kilobase of exon per million fragments mapped. FPKM was used to determine the differential expression of the transcripts*.

**Table 2:** **AD-related genes identified after treatment with GRb1**.

**Gene**	**Transcript ID**	**SAMP8 FPKM**	**SAMP8+Rb1 FPKM**	**SAMR1 FPKM**
Bche	ENSMUST00000029367	0.98	1.27	1.30
Kat2a	ENSMUST00000103118	1.24	2.24	2.50
Dusp1	ENSMUST00000025025	9.07	16.56	16.53
Cdkn2b	ENSMUST00000097981	0.29	0.54	0.62
Fos	ENSMUST00000021674	11.40	20.61	17.49
Glp1r	ENSMUST00000114574	0.27	0.48	0.77
Nr4a1	ENSMUST00000023779	17.55	27.11	25.48
Calr	ENSMUST00000003912	196.95	171.07	163.60
Mapk11	ENSMUST00000088823	14.36	11.96	11.81
Mme	ENSMUST00000029400	0.80	1.48	1.62
Smad3	ENSMUST00000034973	20.09	16.09	16.99
Pdia6	ENSMUST00000057288	85.99	67.21	68.01
Pdia4	ENSMUST00000077290	36.14	26.71	29.83
Xbp1	ENSMUST00000063084	55.75	43.00	42.90
Syk	ENSMUST00000055087	0.73	0.33	0.53
Htr3a	ENSMUST00000003826	4.05	3.33	3.20
Pfkfb3	ENSMUST00000028114	5.22	3.19	4.07
Htr5b	ENSMUST00000055884	0.85	0.46	0.48

*FPKM, fragments per kilobase of exon per million fragments mapped. FPKM was used to determine the differential expression of the transcripts*.

## Discussion

The first detailed record of the medicinal use of *Panax notoginseng* was indicated in the Compendium of Materia Medica, which was written by Li Shizhen and published in A.D. 1596 (Chinese Ming Dynasty) (Zhang and Fang, 2004). PNS, the active ingredient extracted from the root of *Panax notoginseng* (Burk.) F.H. Chen (Araliaceae), has been extensively used to treat various diseases. Li et al. reported that PNS injections are widely applied in clinical situations in China (Li et al., 2009). GRg1 and GRb1, as known members of PNS, attract great attention, especially in reducing the risk of neurodegenerative diseases, such as AD (Wang et al., 2011; Kim et al., 2014; Zhao et al., 2014; Li et al., 2015). However, we believe that staying at this level is far from enough. RNA sequencing (Ozsolak and Milos, 2011), a modern technology, allows for the investigation of the AD-resistant effects of GRg1 and GRb1 in unprecedented detail. In this study, the 7-month-old SAMP8 mice developed severe deficits in learning and memory, and the MWM experiment confirmed this result. SAMP8 mice at this age are relatively “old” in their average life span of 12 months. Meanwhile, the SAMP8 strain is a naturally derived model and shares cognitive, behavioral, and neuropathological alterations observed in aged humans; it can be considered an appropriate animal model to understand the pathogenic mechanisms of AD, especially sporadic AD (Cheng et al., 2014; Kang et al., 2014). However, the transgenic mouse models of AD, such as 3xTg-AD, may simply represent the uncommon familial AD. The primary goal of this research was to systematically analyze the mRNA profiles of the brain of SAMP8 mice after 4 months of GRg1 and GRb1 administration through RNA sequencing. The results indicate that GRg1 and GRb1 can help restrict the development and progression of AD.

First, we identified a total of 74,885 mRNAs in SAMP8, SAMP8+GRg1, and SAMP8+GRb1 mice brains by using the Illumina HiSeq 4000 platform. Then, we compared the mRNA expressions of the SAMP8 and SAMP8+GRg1 mice. We also measured the differential mRNA expression between SAMP8 and SAMP8+GRb1 mice. A total of 1,780 dysregulated mRNA transcripts transcripted from 1,714 genes were detected in the SAMP8 vs. SAMP8+GRg1 group. Moreover, 1,066 differentially expressed mRNA transcripts corresponded to the 1,025 genes found in the SAMP8 vs. SAMP8+GRb1 group. Subsequently, qPCR was used to measure the accuracy of RNA sequencing. Eight dysregulated mRNAs were randomly selected for the test. All results were consistent with the RNA-seq data. The qPCR results confirmed that our resultant mRNAs were of high quality. Evidently, this research provides a solid foundation for further exploration.

GO and KEGG pathway analyses were also performed on the coding genes associated with the significant mRNAs to study the possible biological, cellular, and molecular functions of these mRNAs in AD progression. AD is a multifactorial disease, and many theories have been advanced concerning its causes, including neuron loss, Aβ deposition, tau neuropathology, immune system dysfunction, synapse injury, oxidative stress, and mitochondrial dysfunction (Armstrong, 2013). As expected, our analysis showed that GRg1 and GRb1 exert multi-level anti-AD effects; for example, the MAPK signaling pathway, Notch signaling pathway, PI3K-Akt signaling pathway, and GO terms (e.g., GO: 0000188, GO: 0007420, and GO: 0031175) were affected.

To conceptualize these findings, we established reasonable limitations and further refined the closest results. After treatment with GRg1, we identified 6 upregulated genes, namely, *Bche, Fgfr1, Dusp1, Cdkn2b, Flna*, and *Glp1r*, and 12 downregulated genes, namely, *Slc39a7, Calr, Mapk11, Trpc6, Smad3, Pdia6, Pdia4, Xbp1, Arntl, Gm10053, Pfkfb3*, and *Htr5b*. Meanwhile, in the GRb1 experimental group, 8 genes (*Bche, Kat2a, Dusp1, Cdkn2b, Fos, Nr4a1, Mme*, and *Glp1r*) were up-regulated, and 10 genes (*Calr, Mapk11, Smad3, Pdia6, Pdia4, Xbp1, Pfkfb3, Syk, Htr3a*, and *Htr5b*) were downregulated. Interestingly, 12 genes, including 4 upregulated genes (*Bche, Dusp1, Cdkn2b*, and *Glp1r*) and 8 downregulated genes (*Calr, Mapk11, Smad3, Pdia6, Pdia4, Xbp1, Pfkfb3*, and *Htr5b*) were affected by both GRg1 and GRb1. All of the abovementioned genes showed an expression similar to that in the normal mice (SAMR1). In addition, these genes are highly relevant to AD. Earlier in the article, we discussed *Mapk11* (Stein et al., 1997). Neprilysin, which is encoded by *Mme*, is one of the most important Aβ-degrading enzymes in the prevention of AD pathology (Miners et al., 2012). *Trpc6*, a transient receptor potential canonical (TRPC) related gene, has been reported as the molecular entity associated with Ca^2+^ entry activity (Zhu et al., 1996) and may participate in the AD process. Brain development is impaired in fos –/– mice (Velazquez et al., 2015). As observed, GRg1 and GRb1 are effective Chinese medicines that can be used to prevent and treat AD with multi-system, multi-target, and multi-directional comprehensive regulation. However, assessment of the curative effects of GRg1 and GRb1 merits further investigation in large-scale, clinical studies. This endeavor is going to be an enormous challenge in the future.

In conclusion, we investigated SAMP8 mice brain mRNAs after 4 months of GRg1 and GRb1 administration. Evidently, intake of GRg1 and GRb1 restored the cognitive function of SAMP8 mouse and exerted robust neuroprotective effects in different ways. This strategy provides an invaluable resource for the clinical application of GRg1 and GRb1 in treating AD.

## Author contributions

Wrote the paper, Conceived and designed the experiments: SZ. Performed the experiments: SZ, DZ and WZ. Analyzed the data: SZ, DZ, HL, HZ, CF and WZ. Contributed reagents/materials/analysis tools: SZ and WZ.

### Conflict of interest statement

The authors declare that the research was conducted in the absence of any commercial or financial relationships that could be construed as a potential conflict of interest.
